# Trend analysis for national surveys: Application to all variables from the Canadian Health Measures Survey cycle 1 to 4

**DOI:** 10.1371/journal.pone.0200127

**Published:** 2018-08-09

**Authors:** Yi-Sheng Chao, Chao-Jung Wu, Hsing-Chien Wu, Wei-Chih Chen

**Affiliations:** 1 Centre de recherche du centre hospitalier de l’Université de Montréal (CRCHUM), Université de Montréal, Montréal, Québec, Canada; 2 Département d'informatique, Université du Québec à Montréal, Montréal, Québec, Canada; 3 Taipei Hospital, Ministry of Health and Welfare, New Taipei city, Taiwan; 4 Department of Chest Medicine, Taipei Veterans General Hospital, Taipei, Taiwan; 5 Faculty of Medicine, School of Medicine, National Yang-Ming University, Taipei, Taiwan; Universidad Miguel Hernandez de Elche, SPAIN

## Abstract

**Background:**

Trend analysis summarizes patterns over time in the data to show the direction of change and can be used to investigate uncertainties in different time points and associations with other factors. However, this approach is not widely applied to national surveys and only selected outcomes are investigated. This study demonstrates a research framework to conduct trend analysis for all variables in a national survey, the Canadian Health Measures Survey (CHMS).

**Data and methods:**

The CHMS cycle 1 to 4 was implemented between 2007 and 2015. The characteristics of all variables were screened and associated to the weight variables. Missing values were identified and cleaned according to the User Guide. The characteristics of all variables were extracted and used to guide data cleaning. Trend analysis examined the statistical significance of candidate predictors: the cycles, age, sex, education, household income and body mass index (BMI). R (v3.2) and RStudio (v0.98.113) were used to develop the framework.

**Results:**

There were 26557 variables in 79 data files from four cycles. There were 1055 variables significantly associated with the CHMS cycles and 2154 associated with the BMI after controlling for other predictors. The trend of blood pressure was similar to those published.

**Conclusion:**

Trend analysis for all variables in the CHMS is feasible and is a systematic approach to understand the data. Because of trend analysis, we have detected data errors and identified several environmental biomarkers with extreme rates of change across cycles. The impact of these biomarkers has not been well studied by Statistics Canada or others. This framework can be extended to other surveys, especially the Canadian Community Health Survey.

## Background

Trend analysis that summarizes the patterns across time has been popularly used in a variety of disciplines, such as business[1], financial market[2], economics[3] and epidemics or mortality[4–7]. Trend analysis helps to estimate the quantities of current or previous events and their variability or uncertainties in different time points. It is also the foundation for prediction and projection after analyzing the significance of time and relationships with other predictors[8–10]. For national surveys, certain trends have been studied to show the progress or deterioration in public health and health care[11]. These trends provide important clues for the healthcare professionals to understand the unmet needs for care and the magnitudes of health problems. The comparison of multiple trends allows us to prioritize the issues and allocate resources[4, 12]. If well conducted, projections can be made to further prepare incoming challenges to health systems[8, 9].

However, there are certain issues arising if taking this approach. First, the adjustment of survey design requires researchers to assign appropriate weights and specify survey sampling units and strata[13]. The identification of the necessary variables requires extra attention and expert knowledge. Second, the adjustment of survey design also limits the options of research tools[14]. The automatic procedures developed for time series data or repeated surveys are not applicable concerning survey design[1]. Linear methods, such as generalized linear models and principal component analysis, remain useful for surveys to generate nationally representative statistics[14, 15].

Third, the access to the data may be restricted. For example, some of the Statistics Canada data products can be accessed only through the Research Data Centres (RDC) for academic researchers, such as the Canadian Health Measures Survey (CHMS)[16]. Physical restrictions may prevent complicated or exhaustive research protocols from being conducted for researchers outside Statistics Canada or other collaborating agencies. Fourth, the outcomes analyzed in national surveys are often limited to individuals’ interests. There are many published studies conducted trend analysis of the CHMS data but only limited numbers of variables are taken as target for analysis, especially hypertension and obesity related factors[17–23]. Even if trends are studied by data holders or affiliated researchers, important issues may remain unanswered. For example, the extensive review of environmental chemicals by Health Canada is not informative because statistics are listed by cycle without testing the significance of time trends or association with other contextual factors[24–26]. This needs to be addressed because effective use or extensive application of trend analysis to national surveys may lead to more efficient biomonitoring[11] and better identification of unexpected disease trends[17].

Four, trend analysis may impose challenges to computing resources[27, 28]. The large numbers of variables in national surveys may limit the use of this method if not well planned. Lastly, there may not be sufficient incentive for academia, especially the researchers mainly funded by research grants, to innovate toward novel objectives in the long run[29]. Trend analysis with national surveys requires exhaustive research on documentation and survey method beforehand. There is no immediate benefit by studying variables other than the outcomes that are related to or can lead to research funding.

To address these issues that may be encountered while conducting trend analysis with national surveys, this study aims to 1) propose a framework of trend analysis for all variables in national surveys developed based on the CHMS data, 2) test the feasibility of trend analysis with all CHMS variables using computing resources available to most researchers, 3) summarize the results of the research framework and compatibility with previous studies, and 4) describe some of the obstacles and issues that may be encountered if applied to other surveys.

## Methods

**Fig 1:**
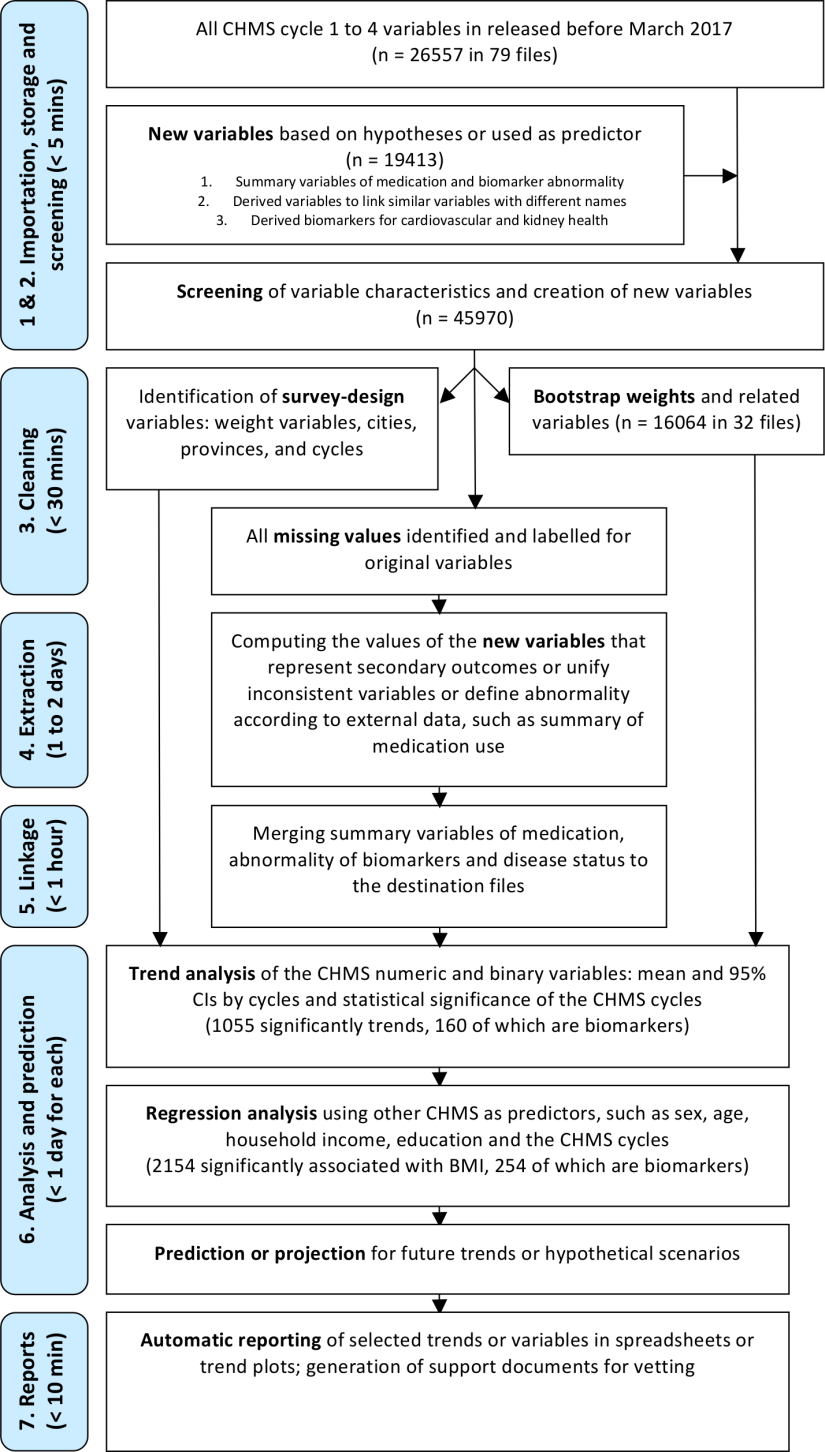
Flowchart of trend analysis with the Canadian Health Measures Survey.

There were several major steps designed to execute this framework with the CHMS data after reviewing the data structure, data dictionaries, the CHMS User Guide[30, 31] and the CHMS Cycle 1 to 8 Content Summary[32]. This framework was applied to the CHMS data to generate a customized research flowchart in Fig 1. First, all variables were imported from data files and screened for basic characteristics, including file names, variables of weights, bootstrap weight files to be merged, maximal and minimal values, responses and variable types (continuous or categorical). For the CHMS variables, the maximal values were important for data cleaning because the missing values were always coded with values far exceeding the observed values[30, 31, 33]. The values ending in 4, 5, 6, 7, 8, and 9 might represent “values higher than limits of detection”, “values less than limits of detection”, “not applicable”, “don’t know”, “refusal” and “not stated”[30, 31, 33]. For other surveys, missing values might be represented with certain values[34] or be coded with reserve values, such as -1 to -3[35]. To prevent computer memory from being exhausted, the data sets were always removed from the memory if unused.

Second, user-defined summary variables were be generated once data was stored for cleaning. The summary variables remained blank at this stage and could be the summaries of medication use, biomarker abnormality, or numbers of chronic conditions, depending on the research objectives. After these two steps, an exhaustive list of the CHMS variables was created. Original and derived variables were listed together and could be important indicators of data processing quality. An illustration of the variable list was shown in Table 1.

**Table 1:** Illustration of the list of variables colored according to sources of data files.

					Types of variable characteristics or statistics						
Variables or types of variables	File name	CHMS Cycle	Weights	Files to retrieve bootstrap weights	1. Basic characteristics	2. Information about survey design	3. Important features for data cleaning, including maximal and minimal values	4. Descriptive statistics to be extracted		5. Results of trend analysis	6. Quality of trend analysis
**ID**	Household data	1	Full weight	…	…	…	…	…		…	…
**Age**	Household data	1	Full weight	…	…	…	…	…		…	…
**Sex**	Household data	1	Full weight	…	…	…	…	…		…	…
**Other original variables**	Household data	1	Full weight	…	…	…	…	…		…	…
**Variables recoded from original variables to derive variables of consistent definitions**	Household data	1	Full weight	…	…	…	…	…		…	…
**Summary variables**	Household data	1	Full weight	…	…	…	…	…		…	…
**ID**	Clinical biomarker	1	Full weight	…	…	…	…	…		…	…
**Other original variables**	Clinical biomarker	1	Full weight	…	…	…	…	…		…	…
**Variables recoded from original variables to derive variables of consistent definitions**	Clinical biomarker	1	Full weight	…	…	…	…	…		…	…
**Summary variables**	Clinical biomarker	1	Full weight	…	…	…	…	…		…	…
**ID**	Environmental chemicals	1	Environmental weight	…	…	…	…	…		…	…
**Other original variables**	Environmental chemicals	1	Environmental weight	…	…	…	…	…		…	…
**Variables recoded from original variables to derive variables of consistent definitions**	Environmental chemicals	1	Environmental weight	…	…	…	…	…		…	…
**Summary variables**	Environmental chemicals	1	Environmental weight	…	…	…	…	…		…	…
**ID**	Fasted samples	1	Fast weight	…	…	…	…	…		…	…
**Other original variables**	Fasted samples	1	Fast weight	…	…	…	…	…		…	…
**Variables recoded from original variables to derive variables of consistent definitions**	Fasted samples	1	Fast weight	…	…	…	…	…		…	…
**Summary variables**	Fasted samples	1	Fast weight	…	…	…	…	…		…	…
**…**	…	1	…	…	…	…	…	…		…	…
**ID**	Household data	2	Full weight	…	…	…	…	…		…	…
**Summary variables**	Household data	2	Full weight	…	…	…	…	…		…	…
**…**	…	1	…	…	…	…	…	…		…	…
**ID**	Clinical biomarker	2	Full weight	…	…	…	…	…		…	…
**…**	…	2	Full weight	…	…	…	…	…		…	…
**…**	…	2	…	…	…	…	…	…		…	…
**ID**	…	3	…	…	…	…	…	…		…	…
**…**	…	3	…	…	…	…	…	…		…	…
**ID**	…	4	…	…	…	…	…	…		…	…
**…**	…	4	…	…	…	…	…	…		…	…

Third, the CHMS data were cleaned based on the reserve values ending in four to nine[30, 31, 33]. The problem particular to biomarker data was that there were values larger or less than the upper or lower limits of detection. Health Canada imputed the values less than the limits of detection with half of the limits of detection[24–26]. In addition, Health Canada excluded the variables with more than 40% of subjects having values less than limits of detection from analysis[24–26]. In contrast, there were currently no official guide to impute values larger than the upper limits of detection and were tentatively imputed with 110% of the upper limits of detection.

Fourth, the summary variables or the derived ones needed to be recoded or calculated after data cleaning. For example, the summary variables of medication use included the use and the numbers of prescription drugs for cardiovascular conditions. This needed to be derived from the drug codes, either Anatomical Therapeutic Chemical (ATC) Classification System or American Society of Health-System Pharmacists (ASHP) drug codes[36]. Another example was that the chronic conditions reported in the CHMS could be further simplified or summarized in the numbers of chronic conditions diagnosed. Abnormality of disease biomarkers could be identified through external information, such as the clinical reference ranges used by health professionals[37, 38]. The numbers of abnormality in biomarkers could be derived after data labeling. Certain secondary biomarkers, such as the estimated creatinine clearance that is used to evaluate kidney health[39, 40], could also be derived after data cleaning.

In addition, some of the original variables needed to be made consistent across the CHMS cycles. The inconsistency arose for a variety of reasons, such as the changes in the measurement sample (serum or plasma), whether subjects fasted or not, and categorization of continuous variables. For example, the level of glucose was measured with plasma in the CHMS cycle 1 and with serum in the other cycles. In cycle 3 and 4, glucose was only quantified with fasted subjects. The glucose measurement with serum or plasma could be taken compatible[41] and could be recoded to the same variable. However, the fasted glucose levels had different diagnostic values from those not fasted and needed to be distinguished[42–44]. Therefore, glucose measured with serum or plasma among fasted and non-fasted subjects were recoded to two variables that represented fasted glucose in cycle 3 and 4 and non-fasted in cycle 1 and 2.

Fifth, some of the summary or derived variables needed to be merged to other data sets to obtain useful statistics. For example, the file of medication use in the CHMS cycle 3 was not assigned survey weights and needed to be merged with the household or other data files to understand issues such as prevalence of drug use or numbers of prescription drugs. The other example was that the information on non-environmental biomarkers in cycle 3 was stored in a stand-alone data set with identification numbers that could be used for data merging. In such cases, the summary variables of medication or abnormality in clinical biomarkers were generated in respective data files and merged to household data files for inference.

Sixth, descriptive or analytical study of all CHMS variables could be conducted. In this study, trend analysis was performed with the CHMS cycles in continuous scales as the only predictor to understand whether there were significantly increasing or decreasing trends across cycles. It was also possible to add more predictors that were important for researchers, such age, sex and provinces. Continuous and binary outcomes were analyzed with linear and logistic regression respectively. The sample sizes, model fit statistics, p values of predictors and variance inflation factors of all predictors were obtained. However, there were several issues to be dealt with for the adjustment of survey design. The sample sizes should be sufficient relative to the primary sampling units. For the CHMS, the sampling units were the cities of clinical visits[30, 31, 33]. The numbers of unweighted sample sizes should satisfy the vetting rules administered by Statistics Canada, which varied by survey and analytical method. The collinearity issue could be assessed between predictors[45]. To avoid memory overload and increase computation efficiency, only necessary variables were loaded for regression analysis. Lastly, the results were reorganized for vetting and release. The trends were plotted against the CHMS cycles along with the necessary summary tables designated for release vetting by the RDC analyst.

Age[46] and blood pressure[47] that had official statistics released were the examples of trend analysis using the CHMS data. The trends were illustrated in relative values compared to the mean values in the CHMS cycle 1. The 95% CIs (confidence intervals) were plotted as shade areas. The details in the blood pressure measurement could be found elsewhere[48, 49]. The significance of time trends was confirmed if there was significant association with the CHMS cycles in continuous scale based on linear regression adjusting for survey design. The association with body mass index (BMI) was also tested with linear regression, while age in years, sex, household income in Canadian dollars, and educations in four categories (less than secondary school education, secondary school education, some post-secondary, and post-secondary graduation) were controlled. BMI was calculated as weight in kilograms divided by height in meters squared[15, 50]. This study was conducted at the Research Data Centre (RDC) at McGill University (Montréal, Québec, Canada). The computer at the RDC was equipped with Intel i7 3070 CPU (central processing unit, 4 cores 8 thread), 16 GB RAM (Random-access memory), 128 GB SSD (solid state disk) and an operating system, Window 7 Professional 64 bit (Microsoft Corporation, Seattle, USA). Data processing and analysis were conducted with R (v3.20)[51] and RStudio (v0.98.113)[52]. Biomarkers were the variables that were identified in the CHMS Cycle 1 to 8 Content Summary[32]. This Summary also defined environmental biomarkers that were the chemicals that could be detected in human specimens or living spaces Statistics Canada, 2015 #451}. P values, two-tailed, less than 0.05 were considered statistically significant. The processing time was reported to help researchers understand the complexity of trend analysis using national surveys.

## Results

### Data processing and analysis

There were 26557 original variables in 79 data files released before March 2017. In 32 data files, 16064 variables were related to bootstrap weights only. There were 19212 variables created to summarize data or derived to represent important secondary outcomes for future projects. Using a typical desktop computer at McGill RDC, the processing time of each major step was estimated in Fig 1. First, the data were imported from STATA format and then stored in R data format. Data importation, storage and screening took less than five minutes to finish. In the third step, the cleaning of all original variables took less than 30 minutes. However, the creation of the summary measures or derived secondary outcomes in the fourth step, such as the numbers of chronic conditions, medication use, and abnormality in biomarkers, was time-consuming. The processing time could be up to two days. At least two factors were contributing to the long processing time. The first factor was that efficient variable-wise calculation was not applicable. Depending on the nature of derived variables, there might be subject-based operation and each observation needed to be screen, for example, for the numbers of cardiovascular or diabetes medication for each individual. The other factor was due to time spent on loading data to memory and writing processed data back to disk.

In the fifth step, the summary or derived variables that needed to be linked to or reproduced in other data files, such as the information on medication use and biomarker summaries, were merged to destination files. For example, the summary of medication use needed to be merged to the household data set and used with appropriate bootstrap weights to obtain nationally representative statistics. This took less than one hour to finish. Sixth, trend or regression analysis with and without the adjustment of other predictors took less than one day to finish for all original or derived variables. The predicted values of all CHMS variables could also be calculated within one day. Lastly, selected trends and summary tables were produced for vetting and release from the RDC within 10 minutes. This research framework took less than four days to screen and analyze all CHMS variables.

### Characteristics of the CHMS cycles and Canadians

The summary of the CHMS data and the population characteristics were shown in Table 2. The cycle 3 had the most numbers of variables and many of them were ever repeated in other cycles. There were cycle-4 variables to be released after April 2017. In cycle 2, there were more biomarkers than in any others. Because of the large numbers of biomarkers in cycle 2 and 3, there were variables designed to label limits of detection for all subjects.

**Table 2:** Characteristics of Canadians and the Canadian Health Measures Survey cycle 1 to 4.

**Cycles**	**Cycle 1**	**Cycle 2**	**Cycle 3**	**Cycle 4**
**Study time**	2007 to 2009	2009 & 2011	2011 to 2013	2014 to 2015
**Number of variables**	6798	6971	9720	3066
**Number of repeated measures**	0	2952	3966	2717
**Number of biomarkers**	325	925	861	201
**Number of variables with limits of detection or quantification**	0	168	176	0
**Weighted N with full weights**	29235444.48	31026646.93	31663898.96	32275596.29
**Proportions of females**	0.502	0.501	0.501	0.501
**Mean ages (years)**	39.3	38.6	39	39.3
**Mean BMI**	26	25.8	25.9	26.2
**Mean household income (Canadian dollars)**	77818.5	80085.7	84779.2	92165.8

**Fig 2:**
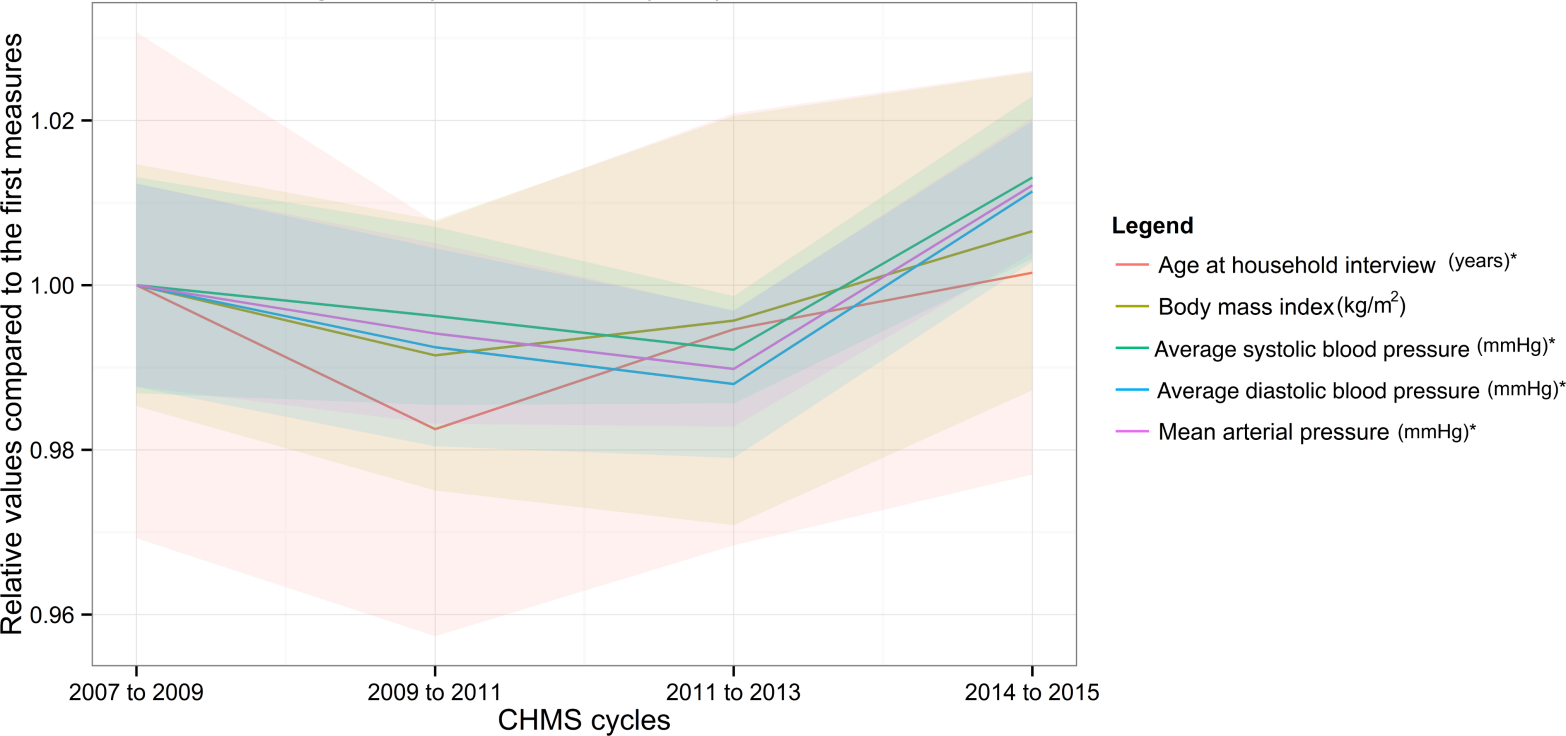
Trends of age and blood pressure based on the Canadian Health Measures Survey. *significantly associated with body mass index (p<0.05).

The numbers of Canadians increased over time, from 29 to 32 million between cycle 1 and 4. About half of them were female. The proportion of females may not be different from that obtained with other data sources[53]. The minimal ages were three years in cycle 1 and six in cycle 2 to 4. The maximal ages were 79 for all cycles. The mean age remained similar and might not be different from the official statistics, which described age by median values[46]. The ranges of blood pressure might also be similar to those published based on the same data[47], while Canadians of all ages were included in this study. In Fig 2, the trends of age, mean arterial pressure, and systolic and diastolic blood pressure were shown along with their 95% confidence intervals (CIs) compared to the first measures in the CHMS. None of the trends was significantly associated with the CHMS cycles (p> 0.05 for all). Age and blood pressure were significantly associated with BMI while controlling for age, sex, education and household income (p <0.05 for all).

### Summary of the trends in the CHMS data

In Table 3, the findings of the trend analysis were summarized. In the first row, the numbers of the CHMS variables that had been repeatedly measured were listed. There were 519 variables measured in CHMS cycle 1 to 4. The rates of change of BMI from cycle 1 to 4 were listed. There were 429 variables significantly associated with the CHMS cycles from one to four and 86 of them were biomarkers identified by Statistics Canada (p<0.05 for all). There were 1099 variables significantly associated with BMI and 152 of them were biomarkers. There were 20 and 26 variables respectively increasing and decreasing for more than 10% in three time intervals from cycle 1 to 4. There were 52 and 68 biomarkers observed to respectively increase or decrease once for at least 10% from cycle 1 to 4. Compared to the average growth rates of BMI, 0.2% per cycle, there were 130 biomarkers increasing more rapidly and 22 of them were non-environmental biomarkers.

**Table 3:** Characteristics of the Canadian Health Measures Survey variables.

**Number of Variables repeated**	**Once**	**2**	**3**	**4**
	4515	2977	978	519
**Number of time intervals**	1	2	3	4
**Relative values of BMI (cycle 1 = 1)**	1	0.991477872	0.995695522	1.006558527
**Number of repeated variables with significant time trends**	429	0	0	0
**Number of repeated biomarkers with significant time trends**	86	0	0	0
**Number of repeated variables significantly associated with BMI**	1099	0	0	0
**Number of repeated biomarkers significantly associated with BMI**	152	0	0	0
**Number of repeated variables significantly associated with prescription use**	542	0	0	0
**Number of repeated biomarkers significantly associated with prescription use**	48	0	0	0
**Number of CHMS variables increase for more than 10% by time intervals**	420	54	20	0
**Number of CHMS variables decrease for more than 10% by time intervals**	385	70	26	0
**Number of biomarkers increase for more than 10% by time intervals**	52	0	0	0
**Number of biomarkers increase for more than 5% by time intervals**	74	5	3	0
**Number of biomarkers decrease for more than 10% by time intervals**	68	2	1	0
**Number of biomarkers decrease for more than 5% by time intervals**	91	6	1	0
**Number of non-environmental biomarkers increase by time intervals**	1	0	0	0
**Number of non-environmental biomarkers decrease for more than 10% by time intervals**	1	1	1	0
**Average changes in BMI per cycle, compared to the first measure**	1.002			
**Number of biomarkers increase more than average growth rate of BMI**	130	19	16	0
**Number of non-environmental biomarkers increase more than average growth rate of BMI**	22	13	16	0
**Prevalence of obesity according to BMI**	0.222	0.236	0.238	0.253
**Prevalence of underweight according to BMI**	0.018	0.024	0.023	0.021

## Discussion

There are large numbers of the CHMS variables and biomarkers increasing or decreasing at high rates. The importance of these trends to public health and wellbeing are not clear because current rate of investigating and publishing the trends of the CHMS variables is not satisfying. There were less than ten trends of the CHMS variables published between 2015 and 2017 including those only considering selected populations[48, 54, 55]. It can take more than ten years to have a comprehensive understanding in the trends of the biomarkers or physical activities or other variables, given the large numbers of variables in national surveys. Currently the CHMS data have been mostly used as a novel data source[12, 18–23, 56, 57], rather than a continuous effort to monitor population health. Only several outcomes have been studied continuously among selected populations[48, 54, 55], in addition to the biomonitoring activities by Health Canada[24–26].

This research framework of trend analysis customized to the CHMS data is highly feasible with computing resources available to most researchers. Scaling up trend analysis to all variables in national surveys has several advantages. In the first place, the automated data cleaning system is effective and efficient. It takes less than 30 minutes to clean all 79 files from the CHMS cycle 1 to 4. The results of data cleaning are examined based on parameters such as the maximal or minimal values to ensure appropriate quality for subsequent trend analysis. Another advantage is that the visualization of trends is easy to understand and useful to prioritize biomarkers or variables for evaluation. In this study, the trends of blood pressure is plotted with the BMI trend to contrast the different patterns. We are applying this method to other variables to find unexpected trends. Moreover, certain types of data errors can also be easily highlighted with the trends. For example, the measurement unit of blood fibrinogen is mislabeled and leads to more than 10-fold decrease in the levels after the CHMS cycle 2 (personal communication with Statistics Canada). The trends with the highest and lowest rates of increase or decrease are easy targets for data quality examination.

Finally, this framework of trend analysis can be supplemented with regression analysis, prediction and projection subsequently. Multiple regression for all CHMS variables to identify the significance of BMI and socioeconomic status has been tried and proven realistic. Predicted values are retrieved to understand the trends least explained by BMI and socioeconomic status (statistics not requested for release). The CHMS has also been used for the projection of obesity trends[10] and projection is also possible.

### Limitations

However, there are several limitations to the research framework. First, there may be other data or documentation errors not identified. The data and documentation accuracy of several of the trends of the largest relative magnitude of change have been confirmed (personal communication with Statistics Canada). There may be other errors that cannot be identified with trend analysis. The other issue is that the imputation method for right- or left-censoring can be improved. Health Canada imputes censored environmental chemicals according to the limits of detection and proportions of subjects within the limits[24, 26, 58]. Other advanced methods may be tried to take other contextual factors into consideration[59, 60]. In fact, it is unclear whether the proportions used by Health Canada are based on weighted or unweighted statistics[24, 26, 58]. This study uses unweighted proportions to exclude the variables from analysis.

Furthermore, the codes have been written inside the RDC and suffered from significant time and resource constraints. The research framework will be structured into an R package for application to other major surveys and research purposes. There are several improvements expected for the implementation. For example, the evaluation of data products can be customized and made interactive. The method to create a list of variable characteristics to be extracted is related to the research hypothesis and should be made flexible for other projects. The introduction of external information to create or derive new variables as predictor or outcome can be improved. We are introducing the reference ranges for clinical or disease biomarkers[37, 38] to further interpret clinical data and population health status. A system that describes the relationships between variables to infer information between them will be useful for sequential questions that study complicated status, such as disease history or evolution of life events. We are also considering incorporating imputation of missing information into the research framework[60].

### Extension to other surveys

This research framework can be extended to other major surveys with similar data structure, variable naming systems, missing value identification strategies and sampling frames, especially the Canadian Community Health Survey[48, 56]. For other major surveys that provide cleaned data[61] or do not use bootstrap weights[35], it requires minimal revision to replicate this research framework to conduct trend analysis for all variables. The automated process for visualization of trend analysis is suggested for researchers to look for neglected trends and for survey administrators to search and correct data errors that can be demonstrated with trends of extreme rates of change across cycles or time points.

## Declaration

### Ethics review

This secondary data analysis was approved by the ethics review committee at the Centre Hospitalier de l’Université de Montréal.

## References

[1] Hyndman RJ, Khandakar Y. Automatic time series for forecasting: the forecast package for R. Monash University, Department of Econometrics and Business Statistics, 2007.

[2] Thomsett MC. A Technical Approach To Trend Analysis: Practical Trade Timing for Enhanced Profits: Pearson Education; 2015.

[3] States United. Internal Revenue Service. Research Division. Trend Analysis and Related Statistics: Department of the Treasury, Internal Revenue Service; 1988.

[4] Chao YS, Boivin A, Marcoux I, Garnon G, Mays N, Lehoux P, et al International changes in end-of-life practices over time: a systematic review. BMC health services research. 2016;16(1):539 Epub 2016/10/08. 10.1186/s12913-016-1749-z PubMed Central PMCID: PMCPMC5048435. 27716238 PMC5048435

[5] NCD Risk Factor Collaboration (NCD-RisC). Trends in adult body-mass index in 200 countries from 1975 to 2014: a pooled analysis of 1698 population-based measurement studies with 19.2 million participants. The Lancet. 2016;387(10026):1377–96. 10.1016/S0140-6736(16)30054-X PMC7615134 27115820

[6] Onwuteaka-Philipsen BD, Brinkman-Stoppelenburg A, Penning C, de Jong-Krul GJ, van Delden JJ, van der Heide A. Trends in end-of-life practices before and after the enactment of the euthanasia law in the Netherlands from 1990 to 2010: a repeated cross-sectional survey. Lancet. 2012;380(9845):908–15. Epub 2012/07/14. 10.1016/S0140-6736(12)61034-4 . 22789501

[7] Cutler JA, Sorlie PD, Wolz M, Thom T, Fields LE, Roccella EJ. Trends in hypertension prevalence, awareness, treatment, and control rates in United States adults between 1988–1994 and 1999–2004. Hypertension. 2008;52(5):818–27. 10.1161/HYPERTENSIONAHA.108.113357 18852389

[8] Wild S, Roglic G, Green A, Sicree R, King H. Global prevalence of diabetes: estimates for the year 2000 and projections for 2030. Diabetes Care. 2004;27(5):1047–53. Epub 2004/04/28. 15111519 10.2337/diacare.27.5.1047

[9] Murray CJ, Lopez AD. Alternative projections of mortality and disability by cause 1990–2020: Global Burden of Disease Study. Lancet. 1997;349(9064):1498–504. Epub 1997/05/24. 10.1016/S0140-6736(96)07492-2 9167458

[10] Twells LK, Gregory DM, Reddigan J, Midodzi WK. Current and predicted prevalence of obesity in Canada: a trend analysis. CMAJ open. 2014;2(1):E18–E26. 10.9778/cmajo.20130016 25077121 PMC3985909

[11] Committee to Review the Department of the Interior's Biomonitoring of Environmental Status and Trends Program, Board on Environmental Studies and Toxicology. A Review of the Biomonitoring of Environmental Status and Trends Program: The Draft Detailed Plan Committee to Review the Department of the Interior's Biomonitoring of Environmental Status and Trends Program, editor. Washington, D.C.: National Academy Press; 1995.

[12] Haines DA, Murray J. Human biomonitoring of environmental chemicals—Early results of the 2007–2009 Canadian Health Measures Survey for males and females. International Journal of Hygiene and Environmental Health. 2012;215(2):133–7. 10.1016/j.ijheh.2011.09.008 22001329

[13] Statistics Canada. Survey Methods and Practices. Statistics Canada, editor. Ottawa, ON: Statistics Canada,; 2010.

[14] Lumley T. Analysis of complex survey samples. Journal of Statistical Software. 2004;9(1):1–19.

[15] Chao Y-S, Wu C-J. Principal component-based weighted indices and a framework to evaluate indices: Results from the Medical Expenditure Panel Survey 1996 to 2011. PLoS ONE. 2017;12(9):e0183997 10.1371/journal.pone.0183997 PubMed PMID: PMC5590867. 28886057 PMC5590867

[16] The Quebec Inter-University Centre for Social Statistics (CIQSS). Statistics Canada Data Montreal, QC: The Quebec Inter-University Centre for Social Statistics (CIQSS),; 2017 [cited 2017 Feb 1]. Available from: https://www.ciqss.org/en/statistics-canada-data.

[17] Ostchega Y, Dillon CF, Hughes JP, Carroll M, Yoon S. Trends in Hypertension Prevalence, Awareness, Treatment, and Control in Older U.S. Adults: Data from the National Health and Nutrition Examination Survey 1988 to 2004. Journal of the American Geriatrics Society. 2007;55(7):1056–65. 10.1111/j.1532-5415.2007.01215.x 17608879

[18] Rawn DFK, Ryan JJ, Sadler AR, Sun W-F, Weber D, Laffey P, et al Brominated flame retardant concentrations in sera from the Canadian Health Measures Survey (CHMS) from 2007 to 2009. Environment International. 2014;63:26–34. 10.1016/j.envint.2013.10.012 24246239

[19] Fisher M, Arbuckle TE, Wade M, Haines DA. Do perfluoroalkyl substances affect metabolic function and plasma lipids?—Analysis of the 2007–2009, Canadian Health Measures Survey (CHMS) Cycle 1. Environmental Research. 2013;121:95–103. 10.1016/j.envres.2012.11.006 23266098

[20] Da Costa LA, Arora P, Garcia-Bailo B, Karmali M, El-Sohemy A, Badawi A. The association between obesity, cardiometabolic disease biomarkers, and innate immunity-related inflammation in Canadian adults. Diabetes Metab Syndr Obes. 2012;5:347–55. Epub 2012/10/12. dmso-5-347 [pii]. PubMed Central PMCID: PMC3468056. 10.2147/DMSO.S35115 23055759 PMC3468056

[21] Rawn DFK, Ryan JJ, Sadler AR, Sun W-F, Haines D, Macey K, et al PCDD/F and PCB concentrations in sera from the Canadian Health Measures Survey (CHMS) from 2007 to 2009. Environment International. 2012;47:48–55. 10.1016/j.envint.2012.05.008 22750796

[22] Colley RC, Garriguet D, Janssen I, Craig CL, Clarke J, Tremblay MS. Physical activity of Canadian adults: accelerometer results from the 2007 to 2009 Canadian Health Measures Survey. Health Rep. 2011;22(1):7–14. Epub 2011/04/23. . 21510585

[23] Bryan S, Saint-Pierre Larose M, Campbell N, Clarke J, Tremblay MS. Resting blood pressure and heart rate measurement in the Canadian Health Measures Survey, cycle 1. Health Rep. 2010;21(1):71–8. Epub 2010/04/30. 20426229

[24] Canada Health. Third Report on Human Biomonitoring of Environmental Chemicals in Canada. Ottawa, ON: Health Canada,; 2015 Available from: http://www.hc-sc.gc.ca/ewh-semt/alt_formats/pdf/pubs/contaminants/chms-ecms-cycle3/chms-ecms-cycle3-eng.pdf.

[25] Health Canada. Second Report on Human Biomonitoring of Environmental Chemicals in Canada. Health Canada, editor. Ottawa, Ontario: Health Canada,; 2013.

[26] Health Canada. Report on Human Biomonitoring of Environmental Chemicals in Canada. Ottawa, ON: Health Canada,; 2010 Available from: http://www.hc-sc.gc.ca/ewh-semt/alt_formats/hecs-sesc/pdf/pubs/contaminants/chms-ecms/report-rapport-eng.pdf.

[27] Maurya MR, Rengaswamy R, Venkatasubramanian V. Fault diagnosis using dynamic trend analysis: A review and recent developments. Engineering Applications of Artificial Intelligence. 2007;20(2):133–46. 10.1016/j.engappai.2006.06.020.

[28] Maurya MR, Rengaswamy R, Venkatasubramanian V. Fault Diagnosis by Qualitative Trend Analysis of the Principal Components. Chemical Engineering Research and Design. 2005;83(9):1122–32. 10.1205/cherd.04280.

[29] Azoulay P, Graff Zivin JS, Manso G. Incentives and creativity: evidence from the academic life sciences. The RAND Journal of Economics. 2011;42(3):527–54.

[30] Statistics Canada. Canadian Community Health Survey (CCHS) Annual component User guide 2014 and 2013–2014 Microdata files Ottawa, ON: Statistics Canada, 2015.

[31] Statistics Canada. Canadian Health Measures Survey (CHMS) Data User Guide: Cycle 2. Ottawa, ON: Statistics Canada, 2013 4. Report No.

[32] Statistics Canada. Canadian Health Measures Survey (CHMS) Content summary for cycles 1 to 8 In: Statistics Canada, editor. Ottawa, ON: Statistics Canada,; 2015.

[33] Statistics Canada. Canadian Health Measures Survey (CHMS) Data User Guide: Cycle 1. Ottawa, ON: 2011 April. Report No.

[34] Health and Retirement Study, RAND HRS Data File (v.P) public use dataset. In: U01AG009740). PadbtUoMwfftNIoAgnN, editor. Ann Arbor, MI2016.

[35] Chao Y-S, Wu C-J, Chen T-S. Risk adjustment and observation time: comparison between cross-sectional and 2-year panel data from the Medical Expenditure Panel Survey (MEPS). Under review. 2013. 10.1186/2047-2501-2-5 PMC4340859 25825669

[36] Joffres M, Shields M, Tremblay MS, Connor Gorber S. Dyslipidemia prevalence, treatment, control, and awareness in the Canadian Health Measures Survey. Can J Public Health. 2013;104(3):e252–7. Epub 2013/07/05. 23823891 10.17269/cjph.104.3783 PMC6974261

[37] Royal College of Physicians and Surgeons of Canada. Clinical laboratory tests—Reference values In: values Clt-R, editor. 10–2015 ed. Ottawa, ON: Royal College of Physicians and Surgeons of Canada,.

[38] Longo D, Fauci A, Kasper D, Hauser S, Jameson J, Loscalzo J. Harrison's Principles of Internal Medicine, 19th Edition: McGraw-Hill Education; 2015.

[39] Eaton D, Pooler J. Chapter 3. Clearance In: Eaton D, Pooler J, editors. Vander’s Renal Physiology 8ed New York: McGraw-Hill; 2013.

[40] Thomis JA, Soep HH, Hallynck T, Boelaert J, Daneels R, Dettli L. Creatinine clearance, different methods of determination. British Journal of Clinical Pharmacology. 1982;13(2):260–2. 10.1111/j.1365-2125.1982.tb01378.x 7059431 PMC1402014

[41] Frank EA, Shubha MC, D'Souza CJM. Blood Glucose Determination: Plasma or Serum? Journal of Clinical Laboratory Analysis. 2012;26(5):317–20. 10.1002/jcla.21524 22585749 PMC6807582

[42] Association AD. Diagnosis and classification of diabetes mellitus. Diabetes Care. 2012;35 Suppl 1:S64–71. Epub 2012/01/04. doi: 35/Supplement_1/S64 [pii] 10.2337/dc12-s064 PubMed Central PMCID: PMC3632174. 22187472 PMC3632174

[43] Inzucchi SE. Diagnosis of Diabetes. New England Journal of Medicine. 2012;367(6):542–50. 10.1056/NEJMcp1103643 22873534

[44] World Health Organization. Use of Glycated Haemoglobin (HbA1c) in the Diagnosis of Diabetes Mellitus: Abbreviated Report of a WHO Consultation Geneva, Switzerland: World Health Organization, 2011. 26158184

[45] Fox J, Weisberg S, Adler D, Bates D, Baud-Bovy G, Ellison S, et al Package ‘car’. 2016.

[46] Milan A. Age and sex structure: Canada, provinces and territories, 2010 Ottawa: Statistics Canada 2011.

[47] Statistics Canada. Blood pressure of Canadian adults, 2009 to 2011. Ottawa, ON: Statistics Canada, 2012 Contract No.: 82-625-X.

[48] Padwal RS, Bienek A, McAlister FA, Campbell NRC. Epidemiology of Hypertension in Canada: An Update. Canadian Journal of Cardiology. 2016;32(5):687–94. 10.1016/j.cjca.2015.07.734 26711315

[49] Wilkins K, Campbell NR, Joffres MR, McAlister FA, Nichol M, Quach S, et al Blood pressure in Canadian adults. Health Rep. 2010;21(1):37–46. Epub 2010/04/30. 20426225

[50] Chao Y-S, Wu H-C, Wu C-J, Chen W-C. Index or illusions: the case of frailty indices in the Health and Retirement Study. 2017. 10.1371/journal.pone.0197859 PMC6051600 30020923

[51] R Development Core Team. R: A language and environment for statistical computing. Vienna, Austria: R Foundation for Statistical Computing; 2016.

[52] RStudio Team. RStudio: Integrated Development for R. Boston, MA: RStudio, Inc.; 2016.

[53] Urquijo CR, Milan A. Female population. Women in Canada: A Gender-Based Statistical Report Component of Statistics Canada Catalogue No 89-503-X. 2011.

[54] Shi Y, de Groh M, Bancej C. Socioeconomic gradients in cardiovascular risk in Canadian children and adolescents. Health Promotion and Chronic Disease Prevention in Canada: Research, Policy and Practice. 2016;36(2):21–31. PubMed PMID: PMC4910426. 10.24095/hpcdp.36.2.02 PMC4910426 26878491

[55] Carson V, Chaput J-P, Janssen I, Tremblay MS. Health associations with meeting new 24-hour movement guidelines for Canadian children and youth. Preventive Medicine. 2017;95:7–13. 10.1016/j.ypmed.2016.12.005 27923668

[56] Atwood KM, Robitaille CJ, Reimer K, Dai S, Johansen HL, Smith MJ. Comparison of Diagnosed, Self-Reported, and Physically-Measured Hypertension in Canada. Canadian Journal of Cardiology. 2013;29(5):606–12. 10.1016/j.cjca.2012.11.019 23395221

[57] Wong SL, Lye EJ. Lead, mercury and cadmium levels in Canadians. Health Rep. 2008;19(4):31–6. Epub 2009/02/21. 19226925

[58] Health Canada. Second Report on Human Biomonitoring of Environmental Chemicals in Canada. Ottawa, ON: Health Canada, 2013.

[59] Puhani P. The Heckman correction for sample selection and its critique. Journal of economic surveys. 2000;14(1):53–68.

[60] Buuren S, Groothuis-Oudshoorn K. mice: Multivariate imputation by chained equations in R. Journal of statistical software. 2011;45(3):1–67.

[61] RAND Corporation. RAND HRS Data Files, supported by NIA and SSA Santa Monica, CA: RAND Corporation,; 2016 [updated September 2016; cited 2016 Nov 29]. Available from: http://www.rand.org/labor/aging/dataprod/hrs-data.html.

